# PHSkb: A knowledgebase to support notifiable disease surveillance

**DOI:** 10.1186/1472-6947-5-27

**Published:** 2005-08-16

**Authors:** Timothy J Doyle, Haobo Ma, Samuel L Groseclose, Richard S Hopkins

**Affiliations:** 1 Division of Public Health Surveillance and Informatics, Epidemiology Program Office, Centers for Disease Control and Prevention (CDC); Atlanta, Georgia, USA; 2 Bureau of Epidemiology, Florida Department of Health; Tallahassee, Florida, USA

## Abstract

**Background:**

Notifiable disease surveillance in the United States is predominantly a passive process that is often limited by poor timeliness and low sensitivity. Interoperable tools are needed that interact more seamlessly with existing clinical and laboratory data to improve notifiable disease surveillance.

**Description:**

The Public Health Surveillance Knowledgebase (PHSkb™) is a computer database designed to provide quick, easy access to domain knowledge regarding notifiable diseases and conditions in the United States. The database was developed using Protégé ontology and knowledgebase editing software. Data regarding the notifiable disease domain were collected via a comprehensive review of state health department websites and integrated with other information used to support the National Notifiable Diseases Surveillance System (NNDSS). Domain concepts were harmonized, wherever possible, to existing vocabulary standards. The knowledgebase can be used: 1) as the basis for a controlled vocabulary of reportable conditions needed for data aggregation in public health surveillance systems; 2) to provide queriable domain knowledge for public health surveillance partners; 3) to facilitate more automated case detection and surveillance decision support as a reusable component in an architecture for intelligent clinical, laboratory, and public health surveillance information systems.

**Conclusions:**

The PHSkb provides an extensible, interoperable system architecture component to support notifiable disease surveillance. Further development and testing of this resource is needed.

## Background

In the United States, notifiable disease reporting is mandated by state and local regulations. These regulations require medical providers and laboratories to notify state and local public health authorities of persons diagnosed with a reportable condition [1]. A reportable condition is one for which regular, frequent, and timely information regarding individual cases is considered necessary for the prevention and control of the disease. Each state determines which conditions are reportable within its jurisdiction. The Council of State and Territorial Epidemiologists (CSTE) determines which diseases all states will voluntarily report nationally to the federal Centers for Disease Control and Prevention (CDC) [2]. Each year, CDC publishes a summary of notifiable disease activity in the United States [3].

Surveillance case definitions provide uniform criteria for reporting notifiable diseases. Case definitions for nationally notifiable diseases have previously been published in printed copy [4] and are currently maintained on the CDC website [5]. The format of case definitions varies somewhat by condition but often contains information on clinical criteria, laboratory criteria, case classification categories, and criteria for classification. The case definitions are written in text to provide guidance to health providers and surveillance epidemiologists when determining whether or not an individual case meets the criteria for reporting. The case definitions are not derived from a formalized information model and are typically not developed for computational purposes. Their content, however, includes numerous terms found in standard medical vocabularies.

Routine notifiable disease surveillance often suffers from incomplete reporting [6] and poor timeliness [7,8]. New threats of bioterrorism have resulted in increased pressures to improve the sensitivity and timeliness of routine disease surveillance, particularly through the use of improved electronic data interchange. Pilot studies have demonstrated improved surveillance sensitivity and timeliness through electronic reporting of laboratory findings by laboratories to public health agencies [9,10]. Other electronic data (e.g., coded discharge diagnoses or pharmacy dispensing data) have also been used to improve the sensitivity and timeliness of routine notifiable disease surveillance [11,12]. Implementation of these methods often depends on the existence of tables that relate the coded laboratory or clinical findings to the notifiable conditions under surveillance. The systematized nomenclature of medicine (SNOMED) and logical observation identifier names and codes (LOINC) have been identified as important vocabulary standards for constructing these tables [13].

Previous efforts to separately maintain information regarding the diseases that are reportable, the content of case definitions, and mapping tables of coded observations to notifiable diseases, have resulted in a proliferation of disparate, unintegrated spread sheets and documents that are used to support notifiable disease surveillance activities. In 2003, we began to develop a database to integrate these information sources – the Public Health Surveillance knowledgebase (PHSkb™). The long-term goal of the PHSkb is to provide a resource to improve the sensitivity, timeliness, and quality of surveillance data through improved electronic data interchange. To achieve this goal, the notifiable disease domain is expressed by using methods of ontology development and knowledge representation, combined with integration of national vocabulary standards that cover the domain. Whereas these methods of ontology development and knowledge representation have been applied to health information retrieval, clinical information systems, and clinical decision support, their application to public health disease surveillance systems is less established. This paper describes the initial creation of the PHSkb. Further field testing will be needed, however, to determine the impact of such methods on surveillance sensitivity, timeliness, and data quality.

## Construction and content

### Data collection

The knowledgebase scope includes diseases, conditions, or other events that are reportable in one or more reporting jurisdictions in the United States. The reporting jurisdictions (n = 52) are those states or cities that report data weekly to CDC via the National Notifiable Diseases Surveillance System (NNDSS), which includes the 50 U.S. states, New York City, and the District of Columbia. Reportable conditions in each jurisdiction were ascertained from the health department website for each jurisdiction [see Additional file 1].

While all jurisdictions provided a list of reportable conditions on their website, in many instances this information was not prominently displayed and required substantial effort to identify. The median time interval needed to identify reporting requirements by navigating the website was approximately one minute, ranging from less than 15 seconds to more than eight minutes. Identifying requirements often required understanding of the organizational structure of the agency and knowledge of which bureau or division was responsible for posting such information to the website. During a 6-month interval between the time the data were first collected and later reconfirmed, 16 (31%) of 52 jurisdictions had updated or otherwise modified their reportable conditions list, suggesting a dynamic information domain.

### Domain ontology

**Figure 1:**
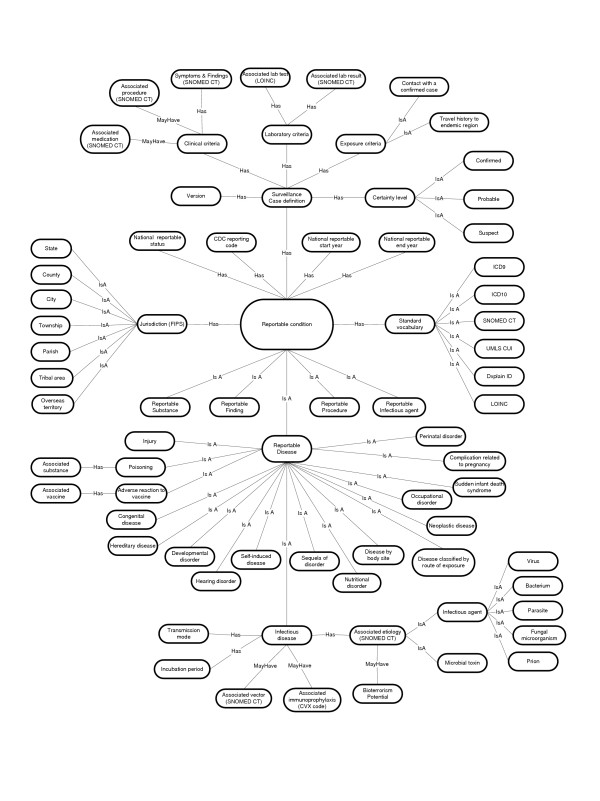
Knowledge representation model of notifiable condition domain.

A knowledge representation model was created for the notifiable disease domain (Figure 1) that depicts the major root concepts in the ontology, their attributes, and relationships between concepts. By necessity, the figure is an oversimplification and does not account for the full hierarchical classification of concepts within the knowledgebase, or identify all the links between concepts. The reader is referred to the PHSkb for the full knowledge representation.

Notifiable conditions include not only single instances of disease, but also infectious agents, substances, procedures, and findings. Reportable findings can include individual findings (e.g., a disease carrier state) or population findings (e.g., an outbreak or cluster of illness). Reportable procedures include concepts such as the administration of medications or prophylaxis specific for a particular disease. Reportable infectious agents include those microbial isolates which laboratories are required to report, independent of clinical illness. For reportable diseases, 18 sub-categories of disease were identified among jurisdiction reporting requirements. However, the majority of reportable diseases fall within the category of infectious disease. For this category, additional attribute knowledge was included, such as the causative agent, insect vector, and associated immunoprophylaxis.

Additional root classes were included for the jurisdictions where events are reportable, and the various terminology standards that cover the domain. Information specified in the surveillance case definition was included as attribute data for each disease and is described in more detail in the content section below.

### Class hierarchy

Instances of the major classes were classified hierarchically, focusing initially on the disease class. The infectious agent, vector, and substance classes were constructed by including all organisms or substances with a causal or other obvious association to concepts in the reportable disease class. Semantic heterogeneity of notifiable events between different reporting jurisdictions was resolved by harmonizing term variants to a standard concept when possible. SNOMED CT (release 1/2003, with the Clue browser) was used extensively to construct the class hierarchy and to harmonize term variations between jurisdictions [14]. In some instances, individual jurisdiction or national data aggregation needs required modification or extension of the concept hierarchy in SNOMED CT. The full list of reportable diseases (n = 373), infectious agents (n = 174), substances (n = 48), findings (n = 58), and procedures (n = 3) is presented in this report along with the number of jurisdictions in which each event is reportable [see Additional files 2 thru 6]. The number of jurisdictions should be interpreted with caution, however, because a particular reportable event might be referred to as a broader or more narrowed concept in another jurisdiction. Furthermore, the reportable condition lists available on the jurisdiction websites, at the time of data collection, might not be the most current reporting requirements given to health providers. The tables are intended to provide a quick glimpse of the breadth of the notifiable condition domain; however, further work is needed to validate the information contained in the knowledgebase with state surveillance partners.

Additional concepts, not reportable in any jurisdiction, were included in the PHSkb to construct the hierarchy for each root class. In the disease class, for example, additional concepts were added either because the condition is a parent-level concept in the class hierarchy under which a reportable disease exists, or the condition is a clinical sub form with distinct clinical symptoms and findings and is already represented by a broader parent concept that is notifiable (i.e., pneumonic and bubonic plague are clinical forms of the notifiable disease plague). More than 570 additional concepts were added to the knowledgebase to construct the class hierarchies.

### Content and relation to vocabulary standards

Concepts in the class hierarchies were mapped to SNOMED-CT and summary results are presented for events reportable in at least one jurisdiction (Table 1). In general, SNOMED-CT provides extensive domain coverage, particularly for the reportable diseases, infectious agents, and substances. Reportable population findings (e.g. disease outbreaks) are not covered as thoroughly in SNOMED. Concepts in the disease class were further mapped to other vocabulary standards. Approximately 76% of concepts in the disease class were in ICD-9 and 48% in ICD-10; 77% had a UMLS concept unique identifier (CUI). Therefore, SNOMED-CT provided better domain coverage than these other coding standards. For the jurisdiction class, Federal Information Processing Standards (FIPS) codes were used to identify each jurisdiction [15].

**Table 1:** Domain content coverage of reportable events with SNOMED CT.

**Semantic class**	**SNOMED CT Content coverage**
Disease	323 of 373 (87%)
Infectious agent	168 of 174 (97%)
Substance	45 of 48 (94%)
Reportable findings	11 of 58 (19%)
Reportable procedures	1 of 3 (33%)

Additional attribute data for each class instance were then entered into the database. When a reportable disease, infectious agent, or substance has been identified as a possible bioterrorism agent or condition [16] the category of BT agent (A, B, or C) was specified in the knowledgebase. Knowledge pertaining to the disease class is the most fully developed in the PHSkb. It contains data on whether or not the disease is nationally notifiable, the year it first became notifiable, and the CDC-assigned code used by jurisdictions to report cases to CDC. For each infectious disease, the associated infectious agents, insect vectors, and incubation periods were identified (when not obvious) either from the surveillance case definition [5] or from the Control of Communicable Diseases Manual [17]. Associated immunoprophylaxes were coded by using the CVX-Vaccines Administered codes developed by CDC for use in immunization registries and adopted by HL7 as a standard code set [18]. Approximately 100 CVX-coded vaccines were associated with 68 reportable diseases in the knowledgebase. The surveillance case definition text was used to populate the clinical and laboratory criteria [5]. Terms from the case definition were parsed and mapped to SNOMED-CT concepts to populate the symptoms & findings, associated procedure, and associated medication slots. A total of 344 findings, 46 procedures, and 19 medications from SNOMED-CT were mapped to terms included in, or implied by, the case definition text.

Finally, the existing table created to support electronic laboratory reporting, that relates LOINC coded laboratory tests to notifiable diseases [19] was imported into the database and used to populate the associated laboratory test field for each disease. We did not attempt to precisely map the laboratory criteria from case definitions to LOINC. Experience has demonstrated that fully specified LOINC terms are often substantially more granular than criteria specified in surveillance case definitions. In addition, the lack of a hierarchical representation within LOINC causes mapping to the less granular case definition criteria to be difficult [20]. However, more that 3,500 LOINC codes have direct relevance as diagnostic tests for reportable diseases and are included in the knowledgebase.

### Development environment

The model was instantiated by using the Protégé-2000 ontology and knowledgebase editing software (version 1.9)[21]. Protégé is an open-source, Java tool that provides an extensible architecture for creating customized knowledge-based applications. Multiple plug-ins have been developed to extend the functionality of Protégé, including various inference and reasoning tools. We used JESS (Java Expert System Shell) tab plug-in (version 1.1) to query the PHSkb[21].

## Utility

The PHSkb has at least three potential uses: 1) providing a framework for the development and maintenance of a controlled vocabulary for reportable events of public health importance; 2) providing convenient, queriable domain knowledge to surveillance epidemiologists, data reporters, and others; and 3) providing a reusable domain knowledge component for intelligent surveillance information system architectures. Each of these broad areas of utility is discussed further in this report.

### Development and maintenance of a controlled vocabulary for reportable conditions

To support the activities of the NNDSS, CDC maintains an authoritative code set for use when jurisdictions report notifiable diseases to CDC. This code set is maintained as a spreadsheet and distributed annually to states when changes occur [22]. This authoritative code set is not concept-based, does not express hierarchical relationships between terms, and focuses predominantly on those conditions that are nationally notifiable.

Our review of the jurisdiction specific websites [see Additional file 1] identified extensive semantic heterogeneity between jurisdictions when referring to reportable conditions, particularly for those conditions that are not nationally notifiable. Conditions reported locally within a jurisdiction that are not nationally notifiable are usually assigned a code by each jurisdiction for use within their system. Without a hierarchical representation, it is difficult to aggregate data across multiple jurisdictions for these diseases, because different jurisdictions use different levels of granularity when defining their own disease reporting requirements. Therefore, aggregation across jurisdictions requires extensive mapping and harmonization of jurisdiction-specific extensions to the code set for notifiable conditions. Having the notifiable disease domain organized across jurisdictions in a hierarchical classification will facilitate data aggregation and electronic data interchange across jurisdictions and between parties within jurisdictions.

CDC-assigned reportable disease codes exist for approximately 128 (34%) of the 373 diseases reportable in at least one jurisdiction. For the remaining diseases reportable in at least one jurisdiction but without a standard name or code (i.e. non-nationally notifiable), non-standard codes are assigned by each jurisdiction, making cross jurisdiction data aggregation difficult or impossible and resulting in a fragmented national surveillance approach. The PHSkb attempts to move from an authoritative code set characterized by incomplete domain coverage to managing the notifiable condition domain as a controlled vocabulary. When fully implemented, features of PHSkb would include harmonizing term variants across jurisdictions, assigning nationally standard codes for locally reportable events, expressing the hierarchical relation between notifiable conditions, and maintaining mappings between notifiable conditions and concept equivalents within other widely used coding standards.

### Queriable domain knowledge

The PHSkb provides convenient, queriable domain knowledge for surveillance epidemiologists and other public health partners. The Protégé software has a built-in query development utility that allows users to construct standard queries and save them to a query library. Several standard queries of the PHSkb have been created and saved in the query library. Examples of such queries include the following.

• In what jurisdictions is a particular disease notifiable?

• What are the reportable conditions in a particular jurisdiction?

• What disease is caused by a particular microorganism?

• What diseases have a particular constellation of symptoms mentioned in their surveillance case definition?

• What diseases are transmitted by a particular insect vector?

• What diseases are associated with a particular laboratory test or finding?

In addition, custom queries can be developed using the inference tools in Protégé (e.g., the JESS tab).

A web interface to the PHSkb is needed to provide broad, web-based, public access to the query functions of the knowledgebase. In the interim, while the web interface is being developed, we have received numerous queries as part of our oversight responsibilities for the NNDSS and have used the PHSkb to respond directly to these domain-specific queries. If adequate, ongoing maintenance of the PHSkb exists, the effort needed to access jurisdiction specific requirements will be reduced by the availability of a central, queriable knowledgebase integrating domain information derived from > 50 different agencies. Such an integrated, centrally-accessed database could be particularly useful to data providers who report to multiple jurisdictions (e.g., large reference laboratories or regional and national provider networks serving communities in different states).

### Reusable architecture component

The PHSkb can function as a reusable component in an architecture for intelligent public health surveillance and clinical information systems [23]. Private software developers have referenced the reporting requirements specified in the PHSkb when developing public health surveillance information systems, and it has been embedded in the architecture of at least one state-based system under development [24]. For users of this state web-based system, the class hierarchies could be navigated and related knowledge could be viewed within the system. When a particular disease was selected for reporting, the system was able to query the knowledgebase and dynamic data entry screens were generated, based on its content. In this way, domain knowledge can be maintained separately by subject matter experts without requiring extensive hard-coding changes to the surveillance software resulting from emerging public health threats or rapidly evolving domain knowledge for a particular disease.

The PHSkb could also be used as an inference engine to identify reportable events from one or more observations. Previous studies have demonstrated that knowledge-based patient screening methods can lead to earlier diagnosis of rare infections, thus improving both clinical patient management and disease surveillance [25]. Two-dimensional tables are currently used to infer cases of reportable disease from LOINC-coded laboratory test observations. In the future, the PHSkb might be able to provide more robust inference capability by integrating 1) laboratory observations with clinical findings and exposure criteria, 2) a hierarchical class structure, and 3) information on jurisdiction-specific requirements. Future development might also address the logic contained in case definitions. Before additional extensions are included, however, further testing is needed regarding the current inference capability of the PHSkb when interacting with laboratory and clinical data streams.

## Discussion

The historical paradigm of notifiable disease surveillance is based on passive reporting of notifiable events from health-care providers to public health agencies. This paradigm relies on public health agencies informing providers of what should be reported (i.e., reportable disease list), a common understanding of the case reporting criteria (i.e., surveillance case definition) and a method for sending and receiving the reports (i.e. telephone hotline, fax, mailed morbidity reports, or web-based reports). Given that there is often minimal reward for reporting or punitive consequences for not reporting, it is not surprising that this passive, unautomated surveillance paradigm often results in poor surveillance sensitivity and timeliness. The information technology and internet revolution during the previous decade has created new opportunities to alter this paradigm and use pre-existing electronic health data to improve the sensitivity and timeliness of surveillance data while reducing the reporting burden on individual providers.

In the same way that clinical practice guidelines have been implemented into rule-based expert systems featuring clinical decision support, the surveillance case definitions provide a basis for developing a rules-based decision support capability for the public health surveillance function [26]. Reusable, extensible domain knowledge components such as the PHSkb are a necessary, but not sufficient, component for fulfilling the paradigm shift from passive disease reporting to efficient, comprehensive, automated electronic data interchange.

Multiple barriers remain, however, for achieving this paradigm shift. First, current reportable disease requirements are unnecessarily fragmented by jurisdiction. The current variability between states regarding reporting requirements makes it cumbersome to develop tools that are generalizable across jurisdictions to assist providers in meeting local reporting requirements. Second, current surveillance case definitions are not based on a uniform information model, are not written for automated interpretation, and often contain ambiguous or conflicting logic. Efforts to retrofit existing case definitions to a standard information model are necessarily awkward and difficult. However, the current definitions do represent an important starting point for standard public health surveillance guidelines, analogous to clinical practice guidelines that often require months or years of consensus-building to create. Third, the use of electronic medical records (EMR) is still not widespread and the cost of implementing EMRs is often a barrier. Finally, more robust clinical vocabulary standards such as SNOMED-CT are not yet widely used in health-care settings. In addition, much of the clinical information contained in patient charts is text that is not electronic and not coded to any vocabulary standard. Further advances are needed in the area of natural language processing and automated methods for converting text data to electronic vocabulary standards.

Despite these important barriers, reusable domain knowledge components such as the PHSkb hold promise for improved interoperability between surveillance information systems and their clinical and laboratory counterparts, through use of a set of integrated content standards for disease surveillance. Field testing of the PHSkb is needed, however, to determine its impact on surveillance metrics such as sensitivity, timeliness, and data quality. Future development of the PHSkb should focus on 1) validating its content with state surveillance partners and subject matter experts, 2) additional development of a queriable web interface to provide broad access to the knowledgebase content and query functions, 3) testing the inference capabilities of the knowledgebase when interacting with clinical and laboratory data streams, and 4) development of an organizational infrastructure and protocols for ongoing maintenance, versioning, and distribution of the knowledgebase. Revisions to the content and structure of the PHSkb should be guided by user feedback and the results of field testing.

## Conclusions

The Public Health Surveillance knowledgebase (PHSkb) provides integrated, extensible domain knowledge regarding notifiable conditions in the United States. It can be used by public health professionals and information system developers to improve the quality of disease surveillance data.

## Availability and requirements

Additional work is needed to validate the PHSkb contents with surveillance partners and subject matter experts. Therefore, the original source documents used to populate the knowledgebase should be regarded as the most definitive source of information regarding the notifiable disease domain. However, the PHSkb is provisionally available for access to demonstrate the methods used and to generate discussion among public health surveillance partners and system developers. The knowledgebase is available for download from the CDC ftp server . The database can be downloaded as three java files suitable for use with the Protégé software (version 1.9 or higher). The total file size is approximately 3 megabytes.

## Abbreviations

CDC Centers for Disease Control and Prevention

CSTE Council of State and Territorial Epidemiologists

CUI concept unique identifier

Dxplain clinical decision support software product

EMR electronic medical record

FIPS Federal Information Processing Standards

FTP File transfer protocol

ICD International Classification of Diseases

JESS Java Expert System Shell

LOINC Logical Observation Identifier Names and Codes

NNDSS National Notifiable Diseases Surveillance System

PHSkb™ Public Health Surveillance Knowledgebase

SNOMED-CT Systematized Nomenclature of Medicine-Clinical Terms

UMLS unified medical language system

URL uniform resource locator

## Competing interests

The author(s) declare that they have no competing interests.

## Authors' contributions

TD conceived of project idea, built knowledgebase, and drafted manuscript text and tables. HM assisted in knowledgebase development, imported data tables, developed custom data queries, contributed to manuscript text, and produced manuscript tables and figures. SG assisted with conceptualization of project, provided initial leadership to obtain project funding, and provided critical review of manuscript text. RH provided broad project management oversight and critical review of manuscript text. All authors read and approved the final manuscript. The findings and conclusions in this report are those of the authors and do not necessarily represent the views of the funding agency.

## Pre-publication history

The pre-publication history for this paper can be accessed here:



## Supplementary Material

Additional File 1 Website addresses for notifiable disease reporting requirements, by jurisdiction Click here for file

Additional File 2 Reportable diseases and number of jurisdictions where reportable Click here for file

Additional File 3 Reportable infectious agents and number of jurisdictions where reportable Click here for file

Additional File 4 Reportable substances and number of jurisdictions where reportable Click here for file

Additional File 5 Reportable findings and number of jurisdictions where reportable Click here for file

Additional File 6 Reportable procedures and number of jurisdictions where reportable Click here for file
