# Supplementary material for: PHSkb: A knowledgebase to support notifiable disease surveillance
Source: BMC Med Inform Decis Mak. 2005 Aug 16;5:27. doi: 10.1186/1472-6947-5-27 (PMC1201144; doi:10.1186/1472-6947-5-27)
Supplement: Additional File 1 — Website addresses for notifiable disease reporting requirements, by jurisdiction [file 1472-6947-5-27-S1.doc]

**Additional file 1. Website addresses for notifiable disease reporting requirements, by jurisdiction***

| **Jurisdiction** | **URL** |
| --- | --- |
| Alabama | <http://www.adph.org/epi/notifdis.pdf>. |
| Alaska | <http://www.epi.hss.state.ak.us/pubs/conditions/crWhat.htm>. |
| Arizona | <http://www.hs.state.az.us/phs/oids/rptlist.htm>. |
| Arkansas | <http://www.healthyarkansas.com/rules_regs/communicable_disease.pdf>.  <http://www.healthyarkansas.com/data/pdf/reportable_disease2004.pdf>. |
| California | [http://www.dhs.ca.gov/ps/dcdc/disb/pdf/PM-110%20CMR%201202.pdf](http://www.dhs.ca.gov/ps/dcdc/disb/pdf/PM-110 CMR 1202.pdf). |
| Colorado | <http://www.cdphe.state.co.us/dc/Medlist.pdf>.  <http://www.cdphe.state.co.us/dc/Lablist.pdf>. |
| Connecticut | <http://www.dph.state.ct.us/BCH/infectiousdise/pdf/2004labrepdislist.pdf>.  <http://www.dph.state.ct.us/BCH/infectiousdise/pdf/2004physrepdislist.pdf>. |
| Delaware | <http://www.state.de.us/dhss/dph/dpc/rptdisease.html>. |
| District of Columbia | <http://dchealth.dc.gov/services/administration_offices/phsa/ddsi/index.shtm>. |
| Florida | <http://www.doh.state.fl.us/disease_ctrl/epi/surv/lor8_4.pdf>.  <http://www.doh.state.fl.us/disease_ctrl/epi/surv/LabList2003.pdf>. |
| Georgia | <http://health.state.ga.us/epi/disease/report.asp>. |
| Hawaii | <http://www.state.hi.us/health/about/rules/11-156.pdf>. |
| Idaho | [http://www.healthandwelfare.idaho.gov/_Rainbow/Documents/health/REPORTDisease%20Poster%20Oct%202004.pdf](http://www.healthandwelfare.idaho.gov/_Rainbow/Documents/health/REPORTDisease Poster Oct 2004.pdf). |
| Illinois | <http://www.idph.state.il.us/health/infect/reportdis.htm>. |
| Indiana | <http://www.in.gov/isdh/publications/comm_dis_rule.pdf>. |
| Iowa | <http://www.idph.state.ia.us/adper/cade_content/epi_manual/4_disease_poster.pdf>. |
| Kansas | <http://www.kdhe.state.ks.us/disease_reporting/download/Ks_disease_report_form.pdf>. |
| Kentucky | [http://chs.ky.gov/publichealth/Programs/Diseases%20&%20Conditions/KYEPID200A.pdf](http://chs.ky.gov/publichealth/Programs/Diseases & Conditions/KYEPID200A.pdf). |
| Louisiana | <http://www.oph.dhh.state.la.us/infectiousdisease/reportdisease/docs/ReportableDiseaseList2003.pdf>. |
| Maine | [http://www.maine.gov/dhs/boh/REPORTABLE%20DISEASES%20IN%20MAINE.pdf](http://www.maine.gov/dhs/boh/REPORTABLE DISEASES IN MAINE.pdf). |
| Maryland | <http://www.edcp.org/pdf/ReportableDisease_HCP_20040223.pdf>.  <http://www.edcp.org/pdf/ReportableDisease_Lab_20040227.pdf>. |
| Massachusetts | <http://www.mass.gov/dph/cdc/epii/reportable/rprtbldiseases_md.pdf>.  <http://www.mass.gov/dph/cdc/epii/reportable/rprtbldiseases_labs.pdf>. |
| Michigan | <http://www.michigan.gov/documents/physicianlistweb_6268_7.pdf>.  <http://www.michigan.gov/mdch/0,1607,7-132-2945_5104-12538--,00.html>. |
| Minnesota | <http://www.health.state.mn.us/divs/idepc/dtopics/reportable/>. |
| Missouri | <http://www.health.state.mo.us/CommunicableDisease/reportablediseaselist2.pdf>. |
| Mississippi | <http://www.msdh.state.ms.us/msdhsite/index.cfm/14,0,194,html>. |
| Montana | <http://www.dphhs.state.mt.us/hpsd/pubheal/disease/pdf/master.pdf>. |
| Nebraska | <http://www.hhs.state.ne.us/cod/HHS-9_(DC).pdf>.  <http://www.hhs.state.ne.us/cod/HHS-10_(DC).pdf>. |
| Nevada | <http://health2k.state.nv.us/Epi/DiseaseNVRept.doc>. |
| New Hampshire | <http://www.dhhs.state.nh.us/DHHS/CDCS/LIBRARY/Policy-Guideline/report-diseases.htm>. |
| New Jersey | <http://www.state.nj.us/health/cd/mdrepdis.pdf>. |
| New Mexico | <http://www.health.state.nm.us/epi/NM_NotifiableConditions_August2003.pdf>. |
| New York | <http://www.health.state.ny.us/nysdoh/cdc/main.htm>.  <http://www.health.state.ny.us/nysdoh/cdc/cdcrept.pdf>. |
| New York City | <http://www.nyc.gov/html/doh/pdf/chi/ltr4-2002.pdf>. |
| North Carolina | <http://www.epi.state.nc.us/epi/gcdc/pdf/10ANCAC41A.pdf>. |
| North Dakota | <http://www.health.state.nd.us/disease/Documents/ReportableConditions.pdf>.  [http://www.health.state.nd.us/disease/Disease%20Reporting/DiseaseCall.htm](http://www.health.state.nd.us/disease/Disease Reporting/DiseaseCall.htm). |
| Ohio | <http://www.odh.state.oh.us/Resources/publications/IDCManual/dcmweb/Intro9.PDF>. |
| Oklahoma | <http://www.health.state.ok.us/program/cdd/noninfec.html>.  <http://www.health.state.ok.us/program/cdd/reportable_disease.htm>. |
| Oregon | <http://www.ohd.hr.state.or.us/acd/mdposter.pdf>.  <http://www.ohd.hr.state.or.us/acd/labpostr.pdf>. |
| Pennsylvania | <http://www.dsf.health.state.pa.us/health/cwp/view.asp?A=171&Q=230520>. |
| Rhode Island | <http://www.health.ri.gov/disease/communicable/summarysheet.pdf>. |
| South Carolina | <http://www.scdhec.net/hs/diseasecont/docs/2004SC_ReportableDiseases.pdf>. |
| South Dakota | <http://www.state.sd.us/doh/Disease/report.htm>. |
| Tennessee | <http://www2.state.tn.us/health/CEDS/notifiable.htm>.  <http://www2.state.tn.us/health/Downloads/ph-1600.pdf>. |
| Texas | <http://www.tdh.texas.gov/ideas/report/default.asp>. |
| Utah | <http://health.utah.gov/els/epidemiology/report.html>. |
| Vermont | <http://www.healthyvermonters.info/hs/epi/idepi/reportable/reportablephysician2004.pdf>.  <http://www.healthyvermonters.info/hs/epi/idepi/reportable/Reportablelaboratory2004.pdf>. |
| Virginia | <http://www.vdh.state.va.us/epi/list.asp>. |
| Washington | <http://www.doh.wa.gov/Notify/default.htm>. |
| West Virginia | [http://www.wvdhhr.org/bph/oehp/sdc/a-z/a-z-idep.htm#Disease%20%20Reporting](http://www.wvdhhr.org/bph/oehp/sdc/a-z/a-z-idep.htm#Disease%20%20Reporting). |
| Wisconsin | <http://www.legis.state.wi.us/rsb/code/hfs/hfs145_app_a.pdf>.  <http://dhfs.wisconsin.gov/dph_bcd/ReportableDiseases/>. |
| Wyoming | <http://wdhfs.state.wy.us/epiid/reportlist.PDF>. |

* Reporting requirements and website addresses in some jurisdictions may have changed during the time the PHSkb was developed. These website addresses were updated on January 4, 2005 in an effort to provide the most current link. However, reporting requirements indicated in the PHSkb may differ from those indicated at these websites, due to changes since the time the information was originally collected.
